# Positive association between actinic keratosis and internal malignancies: a nationwide population-based cohort study

**DOI:** 10.1038/s41598-021-99225-9

**Published:** 2021-10-05

**Authors:** Young Bok Lee, Ji Hyun Lee, Yeong Ho Kim, Ji Min Seo, Dong Soo Yu, Yong Gyu Park, Kyung Do Han

**Affiliations:** 1 Department of Dermatology, Seoul St. Mary’s Hospital, College of Medicine, The Catholic University of Korea, 222, Banpo-daero, Seocho-gu, Seoul, 06591 Republic of Korea; 2 Department of Statistics and Actuarial Science, Soongsil University, Seoul, Republic of Korea; 3 Department of Biostatistics, College of Medicine, The Catholic University of Korea, Seoul, Republic of Korea

**Keywords:** Cancer, Oncology, Risk factors

## Abstract

Little is known about the comorbidities in actinic keratosis patients. To evaluate the association of actinic keratosis with certain malignancies. All patients with actinic keratosis (n = 61,438) and age- and sex-matched control subjects (n = 307,190) at a 5:1 ratio were enrolled using data from the Korean National Health Insurance Service between the years 2007 and 2014. In subjects with actinic keratosis, overall cancer incidence was higher than that for controls after income level, habitat, diabetes, hypertension, and dyslipidemia were adjusted (Hazard Ratio [HR] = 1.43 [95% confidence interval 1.38–1.47]). The positive association of specific cancers were observed in the following order: skin cancer (HR = 3.43 [2.47–4.75]), oral cavity and pharyngeal cancer (HR = 1.99 [1.57–2.52]), lymphoma (HR = 1.59 [1.28–1.96]), leukemia (HR = 1.35 [1.03–1.77]), prostate cancer (HR = 1.35 [1.21–1.51]), renal cancer (HR = 1.29 [1.02–1.63]), liver cancer (HR = 1.21 [1.09–1.35]), thyroid cancer (HR = 1.20 [1.05–1.38]), and gastric cancer (HR = 1.13 [1.03–1.23]). Although further research on pathologic mechanism is needed, the implications of a positive correlation between actinic keratosis and internal organ malignancies has great significance.

## Introduction

Actinic keratosis (AK) is defined as intraepithelial proliferation of atypical keratinocytes. AK is characterized by rough scaly patches or papules on the sun-exposed skin, such as the face and dorsa of the hands, among the elderly. AK occurs in middle-aged and older fair-skinned subjects and is associated with chronic ultraviolet (UV) exposure^1,2^. Although the incidence of AK among Koreans is not as high as the prevalence among Caucasians, the incidence in Korea has increased over the last decade; the prevalence of AK in Korea were 1.95, 4.00, 9.43, 21.90, and 31.81 per 10,000 persons aged in their 40s, 50s, 60s, 70s, and 80s, respectively^3^. In Australia, 60% of the population over the age of 40 had a diagnosis of AK^4^, and AK incidence increased 160% from 1994 to 2012; by 2020, incidence and treatment cost are forecast to increase by 30% from 2012 levels^5^.

Given its potential for progression to squamous cell carcinoma (SCC), AK is a concern from both clinical and economic perspectives. Because it is impossible to predict if and when AK might progress to carcinoma, thorough treatment of field cancerization is considered necessary to prevent potential progression. AK is a disease with a high treatment cost burden^6^; between 2000 and 2003, 5.2 million subjects visited dermatologic clinics in the United States, and the annual total cost of AK treatment was estimated to be US$920 million^7^.

Chronic UV exposure is one of the most important pathogenic factors for development of AKs. UV-related loss of function mutation in the tumor suppressor gene *TP53* results in impaired cell cycle regulation^8^. UV-induced gain of function mutations in oncogene *H-Ras* and oncogene *KNSTRN* was reported to be a pathologic mechanism of cell proliferation^9^. Martincorena et al*.* found that normal-appearing, sun-exposed skin harbored mutations in *NOTCH1, NOTCH2*, and *TP53*, suggesting that UV exposure creates driver mutations and that multiple cancer genes are under strong positive selection even in physiologically normal skin^10^. Other mutations of AK were *IRF4, MC1R,* and *TYR*^11^. However, the mechanism of progression from AK to SCC is still poorly understood.

In a systematic review, the progression rate of AK to SCC was 0–0.08% per lesion-year^12^. The rate of conversion of individual AK to skin cancer is variously reported from 0.03 to 16%^2,13–17^. Criscione et al*.* reported that 0.6% of patients developed a SCC in the AK field during the first year, rising to 2.6% after 4 years^18^. In a 5-year follow-up study, 65% of SCCs were found in previous AK lesions^18^. In an additional study, the probability of malignant transformation to SCC within 10 years in patients with an average of 7.7 AKs was approximately 10%^14^. Foote et al*.* reported that the incidences of basal cell carcinoma and SCC in adults with AK in the USA were 0.04 and 0.03 per person-year, respectively^19^. Chen et al*.* reported a six-fold increase in risk for skin cancer in people with AK compared with people without AK in the United States^20^.

AK is known to occur in elderly and associated with UV radiation, but there has been no research that conducted on whether to increase risks of other cancers except SCC and basal cell carcinoma. A previous study reported that the risk of internal malignancies was significantly higher in the patients with non-melanoma skin cancer patients compared with controls, including bone, nasal cavity and larynx, oral cavity and pharynx, anal, and cervical cancers^21^. We considered the possibility that AK patients might be associated with incidence of other internal malignancy due to their lifestyle or genetic susceptibilities. Studies based on large populations are needed to evaluate the association between cancers and AK. Therefore, the following study was a large-scale cohort investigation of the real incidence of cancer in AK using the Korea Nationwide Health Insurance System (KNHIS) database.

## Results

### Baseline characteristics of study population

We enrolled 61,438 AK patients and 307,190 age-, sex-, and index year-matched controls in this study; the mean age was 62 years in both groups, and the percentage of men in both groups was also the same, 38.8%. The percentage of Korean citizens in the lowest income level was higher in the control group (25.5%) than it was in the AK group (22.5%). There was no difference between groups in habitat, and there was no significant difference in the proportions of individuals with DM; however, hypertension and dyslipidemia were more prevalent among the AK patients than in the control group (Table 1).

**Table 1:** Comparison of population characteristics between control subjects and patients with actinic keratosis.

	Control subjects (*N* = 307,190)	Actinic keratosis patients (*N* = 61,438)	*P *value
Gender (males)	119,265 (38.8)	23,853 (38.8)	1
Age (mean ± SD, years)	62.0 ± 17.2	62.0 ± 17.2	
**Age**			1
20–39	39,445 (12.8)	7889 (12.8)	
40–64	106,940 (34.8)	21,388 (34.8)	
65–	160,805 (52.3)	32,161 (52.3)	
Follow-up years (person-year)	5.00	5.07	1
Lowest income level	78,233 (25.5)	13,796 (22.5)	< 0.01
Place (urban)	134,441 (44.0)	27,088 (44.3)	0.13
Diabetes mellitus	38,890 (12.7)	7791 (12.7)	0.89
Hypertension	108,344 (35.3)	22,017 (35.8)	< 0.01
Dyslipidemia	55,096 (17.9)	13,457 (21.9)	< 0.01

Values are presented as number (%) or mean ± SD.

### Occurrence of all kinds of cancers in AK patients and controls

The occurrence of cancer was significantly higher in the AK group (4420 cancers, 7.19%) than among the controls (15,755 cancers, 5.13%) during the same observation time. The statistically significant differences were for skin, oral cavity, and pharyngeal cancer; lymphoma; leukemia; prostate, renal, liver, thyroid, gastric, and pancreatic cancer; and cancer of the central nervous system (P value less than 0.05, Table 2).

**Table 2:** Comparison of the incidence of cancer between the control and actinic keratosis groups.

	Control groups (*N* = 307,190)		Actinic keratosis (*N* = 61,438)		*P*-value
	Event	%	Event	%	
Number of subjects with any cancers	15,755	5.13	4420	7.19	< 0.01
Skin cancer	89	0.03	61	0.1	< 0.01
Oral cavity and pharyngeal cancer	242	0.08	96	0.16	< 0.01
Lymphoma	337	0.11	108	0.18	< 0.01
Leukemia	252	0.08	68	0.11	0.03
Prostate cancer^†^	1466	0.48	398	0.65	< 0.01
Renal cancer	351	0.11	91	0.15	0.03
Multiple myeloma	200	0.07	26	0.04	0.04
Liver cancer	1742	0.57	423	0.69	< 0.01
Thyroid cancer	1070	0.35	259	0.42	< 0.01
Laryngeal cancer	118	0.04	27	0.04	0.53
Esophageal cancer	215	0.07	49	0.08	0.41
Gastric cancer	2590	0.84	585	0.95	< 0.01
Colorectal cancer	3279	1.07	674	1.1	0.52
Pancreatic cancer	1374	0.45	320	0.52	0.01
Biliary cancer	858	0.28	161	0.26	0.46
Lung cancer	2578	0.84	549	0.89	0.18
Bladder cancer	636	0.21	149	0.24	0.08
Cancer of central nervous system	274	0.09	71	0.12	0.05
Breast cancer*	880	0.29	190	0.31	0.34
Uterine cervical cancer*	251	0.08	51	0.08	0.92
Uterine corpus cancer*	155	0.05	37	0.06	0.33
Ovarian cancer*	283	0.09	61	0.1	0.60
Testicular cancer^†^	29	0.01	5	0.01	0.76

*Female malignancies were analyzed in 187,925 female controls and 37,585 female actinic keratosis patients.

^†^Male malignancies were analyzed in 119,265 male controls and 23,853 male actinic keratosis patients.

### Hazard ratio of all kinds of cancers in AK patients

Table 3 presents the crude IRs for each cancer. After adjustment for income level, habitat, diabetes mellitus, hypertension, and dyslipidemia, AK patients had a marked positive association of overall cancer (HR = 1.43 95% CI [1.38–1.47]), skin cancer (HR = 3.43 [2.47–4.75]), oral cavity and pharyngeal cancer (HR = 1.99 [1.57–2.52]), lymphoma (HR = 1.59 [1.28–1.96]), leukemia (HR = 1.35 [1.03–1.77]), prostate cancer (HR = 1.35 [1.21–1.51]), and thyroid cancer (HR = 1.20 [1.05–1.38]) than the incidence among matched controls. The adjusted HR of multiple myeloma (0.65 [0.43–0.97]) was lower in AK patients than in the controls (Table 3).

**Table 3:** Crude IRs and HRs for overall and specific cancers in AK patients compared with controls with and without adjustment for hypertension, diabetes mellitus, dyslipidemia, habitat and income status.

Cancer	Crude IR per 1000		HR (95% CI) without adjustment	HR (95% CI) with adjustment*
	Actinic keratosis	Controls		
Overall cancer	14.40	10.11	1.43 (1.38, 1.48)	1.43 (1.38, 1.47)
Skin cancer	0.19	0.06	3.43 (2.48, 4.75)	3.43 (2.47, 4.75)
Oral cavity and pharyngeal cancer	0.30	0.15	1.99 (1.57, 2.52)	1.99 (1.57, 2.52)
Lymphoma	0.34	0.21	1.60 (1.29, 1.99)	1.59 (1.28, 1.96)
Leukemia	0.21	0.16	1.35 (1.03, 1.76)	1.35 (1.03, 1.77)
Prostate cancer^¶^	3.31	2.43	1.36 (1.22, 1.52)	1.35 (1.21, 1.51)
Renal cancer	0.28	0.22	1.30 (1.03, 1.63)	1.29 (1.02, 1.63)
Multiple myeloma	0.08	0.12	0.65 (0.43, 0.98)	0.65 (0.43, 0.97)
Liver cancer	1.32	1.09	1.22 (1.09, 1.35)	1.21 (1.09, 1.35)
Thyroid cancer	0.81	0.67	1.21 (1.06, 1.39)	1.20 (1.05, 1.38)
Laryngeal cancer	0.08	0.07	1.15 (0.75, 1.74)	1.15 (0.76, 1.75)
Esophageal cancer	0.15	0.13	1.14 (0.84, 1.55)	1.13 (0.83, 1.54)
Gastric cancer	1.83	1.62	1.13 (1.03, 1.24)	1.13 (1.03, 1.23)
Colorectal cancer	2.11	2.05	1.03 (0.95, 1.12)	1.03 (0.94, 1.11)
Pancreatic cancer	1.00	0.86	1.17 (1.03, 1.32)	1.16 (0.97, 1.17)
Biliary cancer	0.50	0.53	0.94 (0.79, 1.11)	0.94 (0.79, 1.11)
Lung cancer	1.71	1.61	1.07 (0.97, 1.17)	1.06 (0.97, 1.17)
Bladder cancer	0.46	0.40	1.17 (0.98, 1.40)	1.17 (0.98, 1.40)
Cancer of central nervous system	0.22	0.17	1.30 (1.00, 1.68)	1.29 (0.99, 1.68)
Breast cancer^†^	0.94	0.88	1.08 (0.92, 1.26)	1.08 (0.92, 1.26)
Uterine cervical cancer^†^	0.26	0.25	1.02 (0.75, 1.37)	1.02 (0.75, 1.37)
Uterine corpus cancer^†^	0.19	0.16	1.19 (0.83, 1.71)	1.21 (0.85, 1.74)
Ovarian cancer^†^	0.31	0.28	1.08 (0.82, 1.42)	1.08 (0.82, 1.42)
Testicular cancer^¶^	0.04	0.05	0.86 (0.33, 2.23)	0.86 (0.33, 2.21)

*IR* incidence rate, *HR* hazard ratio, *CI* confidence interval.

*Adjustment for diabetes mellitus, hypertension, dyslipidemia, habitat, and income status.

^†^Female malignancies were analyzed in 95,570 females.

^¶^Male malignancies were analyzed in 45,800 males.

### Subgroup analysis according to sex and age group

Among females with AK, the incidence rate of overall cancer (HR = 1.61, 95% CI [1.54–1.68]) was higher than the rate among males (HR = 1.25 [1.19–1.32]). The incidence rates of skin (HR = 4.59 [3.03–6.94]), oral cavity and pharynx (HR = 2.30 [1.59–3.33]), stomach (HR = 1.33 [1.17–1.52]), liver (HR = 1.53 [1.31–1.78]), pancreas (HR = 1.25 [1.06–1.47]), lung (HR = 1.21 [1.04–1.40]), and central nervous system (HR = 1.6 [1.13–2.27]) cancers were markedly higher among the female patients with AK. Male AK patients showed significant associations of lymphoma (HR = 1.81 [1.33–2.47]), thyroid cancer (HR = 1.54 [1.14–2.08]), and renal cancer (HR = 1.41 [1.05–1.88]) (Table 4).

**Table 4:** Subgroup analysis of association between internal malignancy and actinic keratosis by sex and age.

Cancer	Sex		Age (years)		
	Male	Female	20–39	40–64	≥ 65
	Adjusted HR (95% CI)*	Adjusted HR (95% CI)*	Adjusted HR (95% CI)*	Adjusted HR (95% CI)*	Adjusted HR (95% CI)*
Overall cancer	1.25 (1.19, 1.32)	1.61 (1.54, 1.68)	1.30 (1.04, 1.62)	1.18 (1.10, 1.27)	1.52 (1.46, 1.58)
Skin cancer	2.12 (1.22, 3.69)	4.59 (3.03, 6.94)	–	2.08 (0.91, 4.76)	3.85 (2.69, 5.51)
Oral cavity and pharyngeal cancer	1.81 (1.33, 2.47)	2.30 (1.59, 3.33)	–	2.22 (1.46, 3.39)	1.92 (1.44, 2.55)
Lymphoma	1.81 (1.33, 2.47)	1.43 (1.06, 1.94)	0.77 (0.18, 3.43)	1.30 (0.85, 2.01)	1.76 (1.37, 2.28)
Leukemia	1.38 (0.96, 1.99)	1.34 (0.90, 1.98)	1.48 (0.31, 7.13)	1.28 (0.73, 2.25)	1.37 (1.01, 1.87)
Prostate cancer	1.35 (1.21, 1.51)	–	–	1.16 (0.89, 1.50)	1.41 (1.25, 1.59)
Renal cancer	1.41 (1.05, 1.88)	1.12 (0.76, 1.64)	1.58 (0.52, 4.85)	1.22 (0.81, 1.83)	1.31 (0.98, 1.75)
Multiple myeloma	0.58 (0.30, 1.12)	0.69 (0.41, 1.17)	–	0.31 (0.10, 0.98)	0.76 (0.49, 1.18)
Liver cancer	1.00 (0.86, 1.16)	1.53 (1.31, 1.78)	0.95 (0.28, 3.27)	0.99 (0.79, 1.23)	1.30 (1.15, 1.47)
Thyroid cancer	1.54 (1.14, 2.08)	1.13 (0.97, 1.32)	1.37 (0.98, 1.93)	1.16 (0.97, 1.39)	1.20 (0.94, 1.55)
Laryngeal cancer	1.11 (0.72, 1.73)	1.51 (0.41, 5.48)	–	1.08 (0.45, 2.62)	1.17 (0.73, 1.88)
Esophageal cancer	1.06 (0.75, 1.50)	1.49 (0.74, 3.03)	–	0.83 (0.41, 1.69)	1.23 (0.87, 1.73)
Gastric cancer	0.98 (0.86, 1.11)	1.33 (1.17, 1.52)	2.41 (1.14, 5.12)	1.12 (0.93, 1.34)	1.12 (1.00, 1.24)
Colorectal cancer	0.99 (0.88, 1.12)	1.06 (0.95, 1.19)	1.52 (0.77, 2.99)	0.97 (0.81, 1.15)	1.04 (0.94, 1.14)
Pancreatic cancer	1.10 (0.88, 1.27)	1.25 (1.06, 1.47)	2.24 (0.78, 6.44)	1.19 (0.92, 1.55)	1.14 (0.99, 1.31)
Biliary cancer	0.97 (0.75, 1.26)	0.92 (0.74, 1.15)	–	0.79 (0.50, 1.26)	0.96 (0.81, 1.16)
Lung cancer	0.99 (0.88, 1.11)	1.21 (1.04, 1.40)	–	1.09 (0.88, 1.34)	1.06 (0.95, 1.18)
Bladder cancer	1.17 (0.96, 1.44)	1.15 (0.81, 1.63)	–	1.17 (0.78, 1.77)	1.17 (0.96, 1.43)
Cancer of central nervous system	1.01 (0.67, 1.50)	1.6 (1.13, 2.27)	0.42 (0.05, 3.19)	1.61 (0.96, 2.69)	1.25 (0.92, 1.71)
Breast cancer	1.71 (0.35, 8.50)	1.08 (0.92, 1.26)	1.25 (0.72, 2.16)	0.99 (0.79, 1.25)	1.15 (0.91, 1.46)
Uterine cervical cancer	–	1.02 (0.75, 1.37)	0.87 (0.30, 2.51)	0.82 (0.47, 1.44)	1.15 (0.79, 1.69)
Uterine corpus cancer	–	1.21 (0.85, 1.74)	1.88 (0.59, 6.02)	1.02 (0.56, 1.85)	1.27 (0.78, 2.06)
Ovarian cancer	–	1.08 (0.82, 1.42)	1.17 (0.44, 3.10)	1.16 (0.70, 1.91)	1.03 (0.72, 1.47)
Testicular cancer	0.86 (0.33, 2.21)	–	–	–	0.96 (0.37, 2.50)

*Adjustment for diabetes mellitus, hypertension, dyslipidemia, habitat, and income status.

In subgroup analysis according to age group, elderly patients over 65 years old had greater incidence rates of overall cancer (HR = 1.52 [1.46–1.58]), skin cancer (HR = 3.85 [2.69–5.51]), oral cavity and pharyngeal cancer (HR = 1.92 [1.44–2.55]), lymphoma (HR = 1.76 [1.37–2.28]), prostate cancer (HR = 1.41 [1.25–1.59]), leukemia (HR = 1.37 [1.01–1.87]), and liver cancer (HR = 1.30 [1.15–1.47]) than did younger AK patients. However, in AK patients aged 20–39, the incidence rate of gastric cancer (HR = 2.41 [1.14–5.12]) was markedly higher than the rate among AK patients older than age 40. AK patients aged 40–64 showed lower multiple myeloma rate (HR = 0.31 [0.10–0.98]) than did other age groups (Table 4).

## Discussion

This large-scale population-based study showed overall and site-specific cancer incidence rate in Korean patients with AK. Our results showed an increased incidence rate of overall cancer, leukemia, lymphoma, oral cavity and pharyngeal cancer, prostate cancer, and thyroid cancer in AK patients compared with controls after adjusted for confounding factors.

Several researchers have reported increased skin cancer risk in AK patients^20,22–24^. Although AK is considered a premalignant lesion to skin cancer, many AKs persist in the same stage or regress, while only a few cases progress into skin cancer. The key pathomechanism of AK’s progression to skin cancer has not been fully understood. Until now, the number of AKs and their areas and severity indices are considered predictors of advancement to SCC. Green et al. reported that patients with more than 15 AKs had a 10–15 times greater risk of SCC than controls^25^. An AK area and severity index over 7 also suggests an increased risk of SCC transformation^26^. In this study, our findings of increased risk of skin cancer in AK patients (HR, 3.43) are consistent with previous results. A recent study reported that non-melanoma skin cancer patients has increased risk of internal malignancies, including bone, nasal cavity and larynx, oral cavity and pharynx, anus, cervix, thorax, esophagus, breast, lung, stomach cancers, thyroid gland and non-Hodgkin's lymphoma^21^.

In this study, the proportions of oral cavity and pharyngeal cancer were significantly higher in AK patients, which could be because oral SCC is related to AK of the mouth, and oral SCC is the most common oral or pharyngeal cancer^27^. Premalignant oral lesions are associated with smoking habit, oral lichen planus, oral submucous fibrosis, discoid lupus erythematosus, and AK of the mouth^28^, which is also caused by actinic damage on the lower lip (actinic cheilitis) and oral mucosa^29^. That common cause of UV exposure in both AK and oral SCC might explain the increased risk of oral cavity and pharyngeal cancer in AK patients.

In this study, we observed a markedly higher incidence rate of lymphoma among AK patients. A few prior investigators reported increased risk for non-Hodgkin’s lymphoma in skin cancer patients^30,31^. Hu et al. found in large population-based studies this increased risk of subsequent non-Hodgkin’s lymphoma in skin cancer patients and an increased risk of skin cancer in patients with non-Hodgkin’s lymphoma^32^. The association between skin cancer and lymphoma is related to immunosuppression, as suggested by associations of human immunodeficiency virus (HIV) infection^33^ and solid organ transplantation with skin cancer^34^. Recently, Engels et al. investigated 55 medical conditions to identify new associations with Non-Hodgkin’s lymphoma and found links with HIV, solid organ transplantation, hepatitis virus, autoimmune conditions, and, interestingly, nonmelanoma skin cancer and AK^35^. The authors reported that only 3 of the 55 medical conditions were associated with increased risk for all five subtypes of non-Hodgkin’s lymphoma, skin cancer, AK, and hemolytic anemia. In this study, we also found increased risk of lymphoma in AK patients.

Although an association between leukemia and AK has not been reported yet, increased skin cancer risk in chronic lymphocytic leukemia was reported, with rates approximately double those in the general population^36^. Therefore, Mulcahy et al. recommended skin cancer monitoring for patients with chronic lymphocytic leukemia^37^. The increased risk of skin cancer in leukemia patients was explained by their immunocompromised state^38^, but we found in this study an increased risk of leukemia in patients with AK compared with controls vice versa. So far, researchers have reported on several cases of leukemia cutis on AK lesions and on one case of leukemic cells observed in AK lesions and diagnosed as acute myeloid leukemia^39^. Investigators also observed infiltration of leukemic cells in AK lesions of leukemic patients^40^. In this study, it is unknown whether leukemic cell was present in the skin of AK patient who were later diagnosed with leukemia. There might have been diagnostic confusion between AK and leukemia cutis in previous reports, but we enrolled AK patients after excluding previous diagnoses of leukemia, and we still identified increased risk of leukemia in AK patients. These associations may reflect a shared predisposition to both AK and leukemia.

In this study, we observed increased incidence rate of prostate, thyroid, renal, liver, and gastric cancers, but until now, neither AK nor skin cancer has been reported to be associated with these specific cancers in the English literature. One possible explanation is that there might be a link of genetic mutations between AK and these specific cancers. Recently, Yousef et al. reported threefold lower expression of human kallikrein gene 10 (*KLK10*) in AKs compared with normal skin from the same patient, and this decreased expression was found in carcinomas of the skin and prostate. The authors observed overexpression of KLK10 in pancreatic and gastric cancers^41^.

Age, male gender, skin type, and cumulative sun exposure are considered independent risk factors for AK^42^, and recently, an association between tall stature and AK has been reported in a Korean population^43^. In fact, human height has been revealed to have an 80% additive genetic contribution^44^; several single nucleoside variations that are associated with the human height trait are reported in genome-wide association studies^45–47^, and tall individuals have been revealed to be susceptible to cancer including malignant melanoma, leukemia, non-Hodgkin lymphoma, rectal cancer^48^, pancreatic cancer^49^, kidney cancer^50^, prostate cancer^51,52^, and breast cancer^53–56^. A possible explanation of a relationship between height and cancer was that the several genetic variants related to the insulin-growth factor signaling pathway are also related to height^57,58^. The previous reports and the present study showed consistent results that the high-risk cancers in tall individuals accorded with high risks of those cancers in AK patients. However, no researchers have elucidated exact pathomechanisms of any links between AK and specific cancers.

There are some limitations in this study. First, it was an epidemiologic study, and therefore, we could not determine any cause-effect relationships. In addition, we did not include in our analyses all the medications or therapies that might influence the development of cancers. We also lacked information on smoking status, drinking habits, obesity, and family history of cancer and thus could not include these variables in the analyses. Despite these limitations, in this large cohort study, we found that AK patients had a higher overall incidence rate of cancer and higher specific incidence rate of leukemia, lymphoma, oral cavity and pharyngeal cancer, prostate cancer, and thyroid cancer. This study has the implications of a positive correlation between actinic keratosis and internal organ malignancies, but studies are needed on any shared pathogenesis of AK and specific cancers.

## Methods

### Data source and study population

The Korean government mandates the KNHIS database, which covers almost 99% of the Korean population (about 51 million). The Health Insurance and Review Agency operates this comprehensive database of all health care utilization information in Korea^59^. The KNHIS database used is publicly available and identity of participants is not revealing. The KNHIS database includes all claims data as well as a registry of rare incurable diseases such as cancer. The database also contains demographics of subjects including outpatient care history, income level, diagnosis and comorbidities based on International Classification of Disease (ICD)-10 codes, prescriptions, and procedures^60^. The present study was approved by the institutional review board of the Korean National Institute for Bioethics Policy (KNHIS-2018-1-443). The study was also approved by the Catholic University of Korea Institutional Review Board (Approved No. UC18ZESI0134). All methods were carried out in accordance with relevant guidelines and regulations. The need of the written informed consent has waived by the ethics committee of institutional review board of the Korean National Institute for Bioethics Policy and the Catholic University of Korea Institutional Review Board.

### Study design

**Figure 1:**
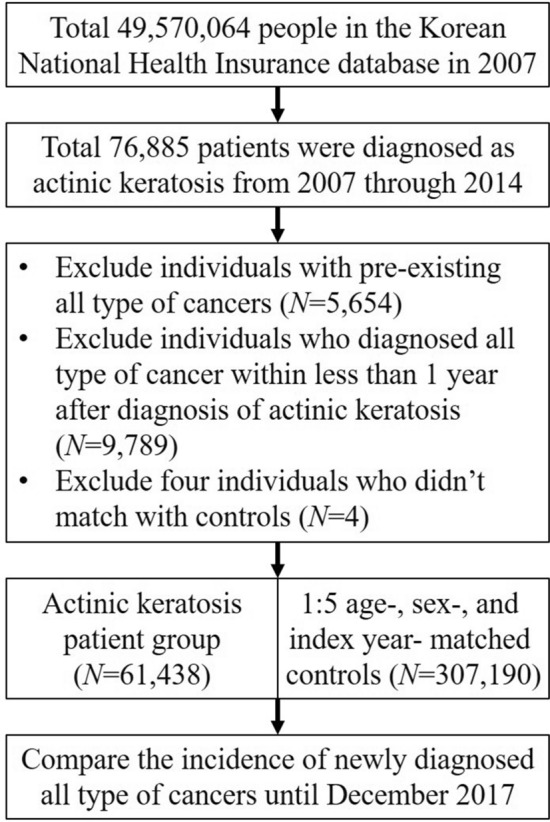
Flow chart of the study design.

The design of this study was similar to that of our previous studies^61,62^. Briefly, we enrolled all the 76,885 subjects who visited clinics or hospitals more than once with an ICD-10 code of AK (L570) in a given year from January 2007 to December 2014 in the KNHIS database. We considered the year in which subjects were first diagnosed with AK to be that subject’s index year. We excluded individuals who already had diagnosed with all type of cancers before the index year (n = 5654) and within a year of the index year (n = 9789) to exclude any preexisting cancers. Four individuals were excluded because we could not find age-, sex-, and index year-matched controls. Ultimately, we analyzed the data of 61,438 subjects with AK and 307,190 age-, sex-, and index year-matched controls without AK, who were randomly selected at a 5:1 ratio, during the same period. Both groups were followed up for cancer development until December 31, 2017 (Fig. 1). We collected the cancer information from the KNHIS database for patients with ICD-10 codes, C00–C96.

### Comorbidities

We needed to adjust for comorbidities such as diabetes mellitus (DM), hypertension, and dyslipidemia that have been reported to increase cancer risks. We considered the presence of DM, hypertension, and dyslipidemia to be established, respectively, by ICD-10 codes E11-14, I10-13 and I15, and E78 with medication.

### Statistical analysis

We calculated the cancer incidence rates (IRs) by dividing the number of incident cases by the total follow-up period presented as 1000 person-years. The censor was defined as individuals who had no cancer until the last day of follow-up (this study is December 31, 2017), or who died before the last day of this study. We used multivariate Cox regression models to assess the cancer incidence rates after we adjusted for confounding factors including income level, habitat, and the comorbidities of DM, hypertension, and dyslipidemia. We defined incidence of overall cancer as a development of any type of cancers in control group and AK group during the follow-up period. And then we analyzed the difference of incidence of each type of site-specific cancer between the AK group and control. In some cases, two types of cancer were registered in the same patient. The claim data have limitations of not being able to determine which cancer is primary or metastatic. In this reason, the competitive risk regression model was not adopted in this study. Overall and site-specific cancer incidences for AK patients and controls are expressed as hazard ratio (HRs) and 95% confidence interval (CIs). We analyzed the study data using SAS version 9.4 (SAS Institute, Cary, NC, USA). A P-value less than 0.05 is considered statistically significant.

## References

[1] Rossi R, Mori M, Lotti T (2007). Actinic keratosis. Int. J. Dermatol..

[2] Ortonne JP (2002). From actinic keratosis to squamous cell carcinoma. Br. J. Dermatol..

[3] Lee JH (2018). Incidence of actinic keratosis and risk of skin cancer in subjects with actinic keratosis: A population-based cohort study. Acta Derm. Venereol..

[4] Frost CA, Green AC, Williams GM (1998). The prevalence and determinants of solar keratoses at a subtropical latitude (Queensland, Australia). Br. J. Dermatol..

[5] Perera E, McGuigan S, Sinclair R (2014). Cost for the treatment of actinic keratosis on the rise in Australia. F1000Res.

[6] Higashi MK, Veenstra DL, Langley PC (2004). Health economic evaluation of non-melanoma skin cancer and actinic keratosis. Pharmacoeconomics.

[7] Warino L (2006). Frequency and cost of actinic keratosis treatment. Dermatol. Surg..

[8] Ziegler A (1994). Sunburn and p53 in the onset of skin cancer. Nature.

[9] Lee CS (2014). Recurrent point mutations in the kinetochore gene KNSTRN in cutaneous squamous cell carcinoma. Nat. Genet..

[10] Martincorena I (2015). Tumor evolution. High burden and pervasive positive selection of somatic mutations in normal human skin. Science.

[11] Jacobs LC (2015). IRF4, MC1R and TYR genes are risk factors for actinic keratosis independent of skin color. Hum. Mol. Genet..

[12] Werner RN (2013). The natural history of actinic keratosis: A systematic review. Br. J. Dermatol..

[13] Glogau RG (2000). The risk of progression to invasive disease. J. Am. Acad. Dermatol..

[14] Marks R, Rennie G, Selwood TS (1988). Malignant transformation of solar keratoses to squamous cell carcinoma. Lancet.

[15] Mittelbronn MA, Mullins DL, Ramos-Caro FA, Flowers FP (1998). Frequency of pre-existing actinic keratosis in cutaneous squamous cell carcinoma. Int. J. Dermatol..

[16] Evans C, Cockerell CJ (2000). Actinic keratosis: Time to call a spade a spade. South Med. J..

[17] Quaedvlieg PJ, Tirsi E, Thissen MR, Krekels GA (2006). Actinic keratosis: How to differentiate the good from the bad ones?. Eur. J. Dermatol..

[18] Criscione VD (2009). Actinic keratoses: Natural history and risk of malignant transformation in the Veterans Affairs Topical Tretinoin Chemoprevention Trial. Cancer.

[19] Foote JA (2001). Predictors for cutaneous basal- and squamous-cell carcinoma among actinically damaged adults. Int. J. Cancer.

[20] Chen GJ (2005). Clinical diagnosis of actinic keratosis identifies an elderly population at high risk of developing skin cancer. Dermatol. Surg..

[21] Yun SJ (2020). Non-melanoma skin cancer as a clinical marker for internal malignancies: A nationwide population-based cohort study. J. Eur. Acad. Dermatol. Venereol..

[22] Philipp-Dormston WG (2018). Patient-reported health outcomes in patients with non-melanoma skin cancer and actinic keratosis: Results from a large-scale observational study analysing effects of diagnoses and disease progression. J. Eur. Acad. Dermatol. Venereol..

[23] Schmitz L, Oster-Schmidt C, Stockfleth E (2018). Nonmelanoma skin cancer—From actinic keratosis to cutaneous squamous cell carcinoma. J. Dtsch. Dermatol. Ges..

[24] Halpern AC, Kopp LJ (2005). Awareness, knowledge and attitudes to non-melanoma skin cancer and actinic keratosis among the general public. Int. J. Dermatol..

[25] Green AC, McBride P (2014). Squamous cell carcinoma of the skin (non-metastatic). BMJ Clin. Evid..

[26] Schmitz L, Gambichler T, Gupta G, Stucker M, Dirschka T (2018). Actinic keratosis area and severity index (AKASI) is associated with the incidence of squamous cell carcinoma. J. Eur. Acad. Dermatol. Venereol..

[27] Lambert R, Sauvaget C, de Camargo Cancela M, Sankaranarayanan R (2011). Epidemiology of cancer from the oral cavity and oropharynx. Eur. J. Gastroenterol. Hepatol..

[28] Warnakulasuriya S, Johnson NW, van der Waal I (2007). Nomenclature and classification of potentially malignant disorders of the oral mucosa. J. Oral Pathol. Med..

[29] Zide MF (2008). Actinic keratosis: From the skin to the lip. J. Oral Maxillofac. Surg..

[30] Levi F, Randimbison L, Te VC, La Vecchia C (1996). Non-Hodgkin's lymphomas, chronic lymphocytic leukaemias and skin cancers. Br. J. Cancer.

[31] Adami J, Frisch M, Yuen J, Glimelius B, Melbye M (1995). Evidence of an association between non-Hodgkin's lymphoma and skin cancer. BMJ.

[32] Hu S, Federman DG, Ma F, Kirsner RS (2005). Skin cancer and non-Hodgkin's lymphoma: Examining the link. Dermatol. Surg..

[33] Silverberg MJ (2013). HIV infection status, immunodeficiency, and the incidence of non-melanoma skin cancer. J. Natl. Cancer Inst..

[34] Euvrard S, Kanitakis J, Claudy A (2003). Skin cancers after organ transplantation. N. Engl. J. Med..

[35] Engels EA (2016). Comprehensive evaluation of medical conditions associated with risk of non-Hodgkin lymphoma using medicare claims ("MedWAS"). Cancer Epidemiol. Biomark. Prev..

[36] Wiernik PH (2004). Second neoplasms in patients with chronic lymphocytic leukemia. Curr. Treat. Options Oncol..

[37] Mulcahy A, Mulligan SP, Shumack SP (2018). Recommendations for skin cancer monitoring for patients with chronic lymphocytic leukemia. Leuk. Lymphoma.

[38] Dong J, Lee T, Desman GT, Ratner D (2020). Risk factors for recurrent and metastatic cutaneous squamous cell carcinoma in immunocompromised patients. J. Am. Acad. Dermatol..

[39] Blattner C, DeDonato A, Blochin E, Kazlouskaya V, Elston DM (2014). Initial presentation of acute myelogenous leukemia in the infiltrate underlying an actinic keratosis. Indian Dermatol. Online J..

[40] Smoller BR, Warnke RA (1998). Cutaneous infiltrate of chronic lymphocytic leukemia and relationship to primary cutaneous epithelial neoplasms. J. Cutan. Pathol..

[41] Yousef GM (2005). Identification of new splice variants and differential expression of the human kallikrein 10 gene, a candidate cancer biomarker. Tumour Biol..

[42] Harvey I, Frankel S, Marks R, Shalom D, Nolan-Farrell M (1996). Non-melanoma skin cancer and solar keratoses II analytical results of the South Wales Skin Cancer Study. Br. J. Cancer.

[43] Lee YB (2018). Association between height and actinic keratosis: A nationwide population-based study in South Korea. Sci. Rep..

[44] Silventoinen K (2003). Heritability of adult body height: A comparative study of twin cohorts in eight countries. Twin Res..

[45] Yang J (2010). Common SNPs explain a large proportion of the heritability for human height. Nat. Genet..

[46] Lango Allen H (2010). Hundreds of variants clustered in genomic loci and biological pathways affect human height. Nature.

[47] Wood AR (2014). Defining the role of common variation in the genomic and biological architecture of adult human height. Nat. Genet..

[48] Emerging Risk Factors Consortium (2012). Adult height and the risk of cause-specific death and vascular morbidity in 1 million people: individual participant meta-analysis. Int. J. Epidemiol..

[49] Aune D (2012). Height and pancreatic cancer risk: A systematic review and meta-analysis of cohort studies. Cancer Causes Control.

[50] Liang S, Lv G, Chen W, Jiang J, Wang J (2015). Height and kidney cancer risk: A meta-analysis of prospective studies. J. Cancer Res. Clin. Oncol..

[51] Farwell WR (2011). The association between height and prostate cancer grade in the Early Stage Prostate Cancer Cohort Study. Cancer Causes Control.

[52] Zuccolo L (2008). Height and prostate cancer risk: A large nested case-control study (ProtecT) and meta-analysis. Cancer Epidemiol. Biomark. Prev..

[53] Benderli Cihan Y (2017). Is height of prognostic significance in breast cancer cases?. Asian Pac. J. Cancer Prev..

[54] Elands RJ (2017). A systematic SNP selection approach to identify mechanisms underlying disease aetiology: Linking height to post-menopausal breast and colorectal cancer risk. Sci. Rep..

[55] Qian F (2019). Height and body mass index as modifiers of breast cancer risk in BRCA1/2 mutation carriers: A Mendelian randomization study. J. Natl. Cancer Inst..

[56] Zhang B (2015). Height and breast cancer risk: Evidence from prospective studies and Mendelian randomization. J. Natl. Cancer Inst..

[57] He M (2015). Meta-analysis of genome-wide association studies of adult height in East Asians identifies 17 novel loci. Hum. Mol. Genet..

[58] Lettre G (2008). Identification of ten loci associated with height highlights new biological pathways in human growth. Nat. Genet..

[59] Song SO (2016). Trends in diabetes incidence in the last decade based on Korean National Health Insurance Claims Data. Endocrinol. Metab. (Seoul).

[60] Koo BK, Lee CH, Yang BR, Hwang SS, Choi NK (2014). The incidence and prevalence of diabetes mellitus and related atherosclerotic complications in Korea: A National Health Insurance Database Study. PloS One.

[61] Lee JH (2019). Cancer risk in 892 089 patients with psoriasis in Korea: A nationwide population-based cohort study. J. Dermatol..

[62] Na SJ (2018). Cancer risk in patients with Behcet disease: A nationwide population-based dynamic cohort study from Korea. J. Am. Acad. Dermatol..

